# Early Neuromuscular Electrical Stimulation and In‐Bed Leg Cycling Preserve Quadriceps Femoris Muscle Thickness in Critically Ill Patients

**DOI:** 10.1002/pri.70113

**Published:** 2025-09-23

**Authors:** Bruna Scherer Lorenzoni, Marina Torres Machado, Maurício Tatsch Ximenes Carvalho, João Pedro Martins de Albuquerque, Helena Biermann Pereira, Rafaela Bassan Bortoluzi, Dannuey Machado Cardoso, Michele Forgiarini Saccol, Tamires Daros dos Santos, Isabella Martins de Albuquerque

**Affiliations:** ^1^ Curso de Fisioterapia Universidade Federal de Santa Maria (UFSM) Santa Maria Rio Grande do Sul Brasil; ^2^ Universidade Federal de Pelotas (UFPEL) Pelotas Rio Grande do Sul Brasil; ^3^ Curso de Medicina Universidade de Santa Cruz do Sul (UNISC) Santa Cruz do Sul Rio Grande do Sul Brasil; ^4^ Centro de Ensino Superior Dom Alberto Santa Cruz do Sul Rio Grande do Sul Brasil; ^5^ Laboratório de Biomecânica Clínica (LaBioC) Departamento de Fisioterapia e Reabilitação Universidade Federal de Santa Maria (UFSM) Santa Maria Rio Grande do Sul Brasil; ^6^ Empresa Brasileira de Serviços Hospitalares (EBSERH)/Hospital Universitário de Santa Maria (HUSM) Santa Maria Rio Grande do Sul Brasil; ^7^ Programa de Pós‐Graduação em Ciências do Movimento e Reabilitação Departamento de Fisioterapia e Reabilitação Universidade Federal de Santa Maria (UFSM) Santa Maria Rio Grande do Sul Brasil

**Keywords:** critical illness, early ambulation, electrical stimulation, intensive care units, muscle strength, quadriceps muscle

## Abstract

**Background and Purpose:**

Muscle atrophy occurs early in critical illness, particularly in the leg muscles, which are most vulnerable to weakness due to immobility in the intensive care unit (ICU). However, the efficacy of early neuromuscular electrical stimulation (NMES) and in‐bed leg cycling added to standardized rehabilitation as a strategy to prevent quadriceps femoris muscle atrophy in critically ill patients remains uncertain. This study aimed to investigate the effects of early NMES and in‐bed leg cycling on quadriceps muscle thickness (QMT) and muscular strength in critically ill patients.

**Methods:**

This prospective quasi‐experimental study included 36 critically ill adult patients within the first 24 h of mechanical ventilation after admission. Patients were allocated into three groups: NMES added to standardized rehabilitation group (*n* = 12), in‐bed cycling exercise added to standardized rehabilitation group (*n* = 12) or standardized rehabilitation group (*n* = 12). The following outcomes were assessed: QMT (ultrasound) and global muscle strength (Medical Research Council—MRC–Sum Score).

**Results:**

The standardized rehabilitation group showed a significant reduction in QMT (*p* = 0.001). The NMES group (*p* < 0.01) and in‐bed cycling group (*p* < 0.001) preserved QMT compared to the standardized rehabilitation group. An increase in global muscle strength was observed in the NMES (*p* < 0.001) and in‐bed cycling groups (*p* < 0.001), although no differences were found among the three groups.

**Discussion:**

Our findings suggest that NMES and in‐bed cycling exercise may be effective in preserving QMT in critically ill patients. Larger trials are required to confirm the efficacy of NMES and in‐bed cycling exercise on muscle mass preservation and improvement in global muscle strength in ICU patients.

## 1 Introduction

Critically ill patients frequently develop early‐onset muscle atrophy, particularly in the leg muscles, which are most vulnerable to weakness due to immobility in the intensive care unit (ICU) (Mart et al. 2025). On average, patients with critical illness lose nearly 2% of skeletal muscle per day during the first week of ICU admission (Fazzini et al. 2023). Quadriceps size can decrease by approximately 18% during a 10‐day ICU stay, with the most significant muscle loss occurring within the first 3 days of admission (Puthucheary et al. 2013), and greater loss of quadriceps muscle thickness (QMT) is associated with worse outcomes (Toledo et al. 2021). In this sense, intensive care unit‐acquired weakness (ICU‐AW) is common in critically ill patients and is characterized by muscle weakness and physical function loss (Fuentes‐Aspe et al. 2024).

Numerous studies have demonstrated that ultrasound is a non‐invasive, reliable and cost‐effective tool to detect muscle mass loss wasting in critically ill patients at the bedside (Toledo et al. 2021; Pardo et al. 2018) and reliability for the measurement of QMT using ultrasound has been demonstrated in individuals with critical illness (Yao et al. 2024). Moreover, ultrasound provides the monitoring of changes in QMT to implement early interventions that reduce acute muscle wasting during critical illness (Nickels, Aitken, Barnett, Walshamet, et al. 2020a; Nickels, Aitken, Barnett, Walsham, et al. 2020b) and has promise as a surrogate measure of skeletal muscle strength in sedated or comatose patients unable to participate in volitional muscle strength assessments. In a recent prospective validation study, Peres et al. (2024) demonstrated a high correlation between the measurement of QMT using ultrasound and computed tomography.

In the last decade, early physical rehabilitation interventions (within 24–72 h after ICU admission) have emerged as a safe and crucial intervention to prevent or mitigate the developing intensive care unit acquired weakness (ICU‐AW), or both, and to improve functional outcomes for patients (Himanshu and Bakhru 2024). In this scenario, in‐bed cycling may facilitate the early initiation of exercise interventions with critically ill patients that can be introduced before patients are able to follow commands (Nickels, Aitken, Barnett, Walshamet, et al. 2020a; Nickels, Aitken, Barnett, Walsham, et al. 2020b). Recently, neuromuscular electrical stimulation (NMES), an alternate mode of nonvolitional rehabilitation intervention that involves sequential activation of muscles, has also been gradually applied in the early mobilization of critically ill patients (Yang et al. 2023).

Muscle thickness, assessed by ultrasound provides an opportunity to detect early muscle mass loss and intervene before ICU‐AW is established (B. Connolly et al. 2015). A recent randomized trial demonstrated that the addition of in‐bed cycling or NMES‐cycling to standard physical therapy did not reduce muscle wasting of the rectus femoris in the ICU and following discharge to the post‐ICU ward (Rollinson et al. 2024). In a randomized controlled trial, our research group demonstrated that early passive in‐bed cycling may assist the recovery of global muscle strength in ICU patients (Machado et al. 2017). Another study involving patients admitted to the ICU investigated the effects of adding early in‐bed leg cycling plus electrical muscle stimulation to a standardized early rehabilitation program and found no improvement in global muscle strength at ICU discharge (Fossat et al. 2018). However, the effects of NMES and in‐bed cycling exercise added to standardized rehabilitation on QMT and muscle strength in critically ill patients remain unclear. Therefore, this study was conducted to investigate the effects of early NMES and in‐bed leg cycling added to standardized rehabilitation on QMT and global muscle strength in critically ill patients.

## 2 Methods

### 2.1 Study Design

A prospective quasi‐experimental study was conducted at the ICU of the Hospital Universitário de Santa Maria (HUSM), Santa Maria, Rio Grande do Sul, Brasil. The setting was a 16‐bed tertiary mixed clinical, surgical and neurological ICU. The study was approved by the ethics committee (process no. 1.067.797) and performed in accordance with the Declaration of Helsinki. All family members provided written informed consent prior to participation.

### 2.2 Participants

All patients recruited were evaluated by electronic health records, demographic characteristics, primary diagnosis at ICU admission, and according to the following clinical scores: Acute Physiology and Chronic Health Evaluation II (APACHE II) and Sequential Organ Failure Assessment (SOFA) Score. Eligible patients were adults (≥ 18 years of age) admitted to the ICU and received at least 24 h of invasive MV within the first 48 h of their ICU stay were eligible. Patients were excluded if they (I) pregnant, (II) cardiac arrest was the cause of ICU admission or had cardiac arrest before screening, (III) stroke (IV) had confirmed or suspected neuromuscular weakness (V) had advanced dementia, (VI) had deep venous thrombosis or pulmonary embolism treated for less than 48 h, (VII) had a pacemaker (VIII) had a contraindication to receive bed cycling or electrical stimulation for musculoskeletal, dermatological, or surgical reasons, (IX) had a lower limb that was amputated, or (X) were unlikely to survive the current hospital admission or had palliative goals of care.

### 2.3 Measurements

#### 2.3.1 Quadriceps Muscle Thickness

The QMT (rectus femoris and vastus intermedius) was assessed with high‐resolution ultrasound (Mindray Ultrasound, portable DP‐2200, China), in B mode, with an echocardiologic transducer (65C15EA 5.0–9.0 MHz, 4 W). The thickness measurements were obtained on the first 24 h of MV (pre‐intervention) and at ICU discharge (post‐intervention). The same operator, blinded to group allocation, who was trained in using bedside ultrasound performed the measures.

Each patient was assessed in the supine position with the knee in passive extension and neutral rotation, as described previously (B. Connolly et al. 2015). A thick layer of ultrasound gel was maintained between the probe‐skin interface to ensure that there was no tissue depression, and the operator ensured that the muscle belly and skin maintained its convex shape prior to freezing the image. Three anterior images were obtained from the patient's right leg. The transducer was positioned perpendicularly on the skin with minimal pressure at the quadriceps femoris midpoint, a region located between the anterior superior iliac spine and the superior pole of the patella. To ensure measurement reproducibility, a suitable site was marked on the patient's skin with a permanent marker. The images were analyzed using ImageJ software (NIH, Bethesda, MD, USA), and the average values were used in the analysis (Fivez et al. 2016). The QMT was calculated as the sum of the thicknesses of the rectus femoris and vastus intermedius thickness and expressed in centimeters (cm).

### 2.4 Global Muscle Strength

Global muscle strength was assessed using the Medical Research Council (MRC) grading system (Medical Research Council of the United Kingdom 1978) to determine the strength of 6 muscle groups on both sides of the body (overall score range, 0–60 points). MRC score ≤ 48 defined ICU‐AW (Latronico and Gosselink 2015). Global muscle strength was evaluated on the first day the patient was awake and cooperative (Richmond Agitation and Sedation Scale [RASS] ≥ −2) and at ICU discharge by an experienced physical therapist using the MRC grading system.

### 2.5 Study Protocol

After baseline evaluations, all patients within the first 24 h of invasive MV were randomly assigned using sealed opaque numbered envelopes to one of three groups: the NMES added to standardized rehabilitation group, in‐bed cycling exercise added to standardized rehabilitation group or to standardized rehabilitation group. For all three groups, the interventions started from the patient's inclusion in the study and until at ICU discharge. The interventions were provided by physiotherapists with experience in critical care rehabilitation.

Standardized rehabilitation group: In the usual care group, standardized early rehabilitation included conventional physical and respiratory therapy for 30 min twice daily, 7 days per week. The protocol included vibrocompression maneuvers, lung hyperinflation by the mechanical ventilator, and tracheal aspiration, when necessary, as well as passive and active‐assisted motor exercises for arms and legs, depending on the clinical course of patients (Machado et al. 2017).

NMES added to standardized rehabilitation group: In addition to standardized early rehabilitation as described above, electric stimulation was performed 30 min per day, 7 days a week (Fischer et al. 2016). The NMES was applied using a calibrated electrical stimulator (Neurodyn High Volt, IBRAMED, São Paulo/SP, Brazil) with four self‐adhesive electrodes (90 × 50 mm; MultiStick, Axelgaard Manufacturing Co Ltd, Fallbrook, CA, USA) placed over the motor points of the quadriceps femoris muscle (two on the rectus femoris, one on the vastus medialis, and one on the vastus lateralis). The electrodes were placed on both legs. The following parameters were used: 50 Hz frequency, pulse duration of 400 microseconds (*μ*s), 12 s off, 6 s on (Kho et al. 2015). The intensity was gradually increased until muscle contraction (confirmed by inspection and palpation). Once the patient was more alert (RASS 0), they encouraged to tolerate as much stimulation as possible during electrical stimulation and should be relaxed during NMES to avoid spontaneous quadriceps contractions and co‐contraction of leg muscles (Y. Liu et al. 2023). In‐bed cycling exercise added to standardized rehabilitation group: In addition to standardized early rehabilitation as described above, patients also received once daily 20 min/day of in‐bed cycling exercise (MOTOmed letto2; RECK‐Technik GmbH & Co. KG, Betzenweiler, Germany) for 7 days/week under the supervision of a trained physical therapist. To conduct early in‐bed cycling exercise, patients were placed in the supine position with their head elevated at 30°. To ensure that the passive exercises were properly carried out, the screen was monitored to visualize the training and detect any active movements. The exercise protocol was conducted in the passive cycling mode for 30 min at a fixed rate of 20 cycles/min (RASS −4 to −1). At awakening (RASS 0), patients were encouraged to progress to active exercise, with intensity graded according to the rate of perceived exertion (between 3 and 5) on the modified Borg Scale (Aquim et al. 2019).

The protocol could be discontinued according to the participant's request or if safety concerns arose. Physiological stability was monitored during the study to ensure the patient's safety, including monitoring peripheral oxygen saturation (SpO_2_), heart rate (HR), mean arterial pressure, and systolic and diastolic blood pressure evaluated with a multiparameter monitor (DX 2022; Dixtal Biomedica, Manaus, Brazil). The following parameters were used as the criteria for discontinuing the protocol: hemodynamic instability (mean arterial pressure < 60 or > 125 mmHg), SpO_2_ < 88%, HR > 130 bpm or < 40 bpm, and signs of respiratory distress.

### 2.6 Sample Size Calculation

The primary endpoint QMT was used to calculate the sample size. The sample size was calculated using GPower (version 3.1.9.7). Based on a pilot study including the first 10 participants and considering an alpha level of 5% and a statistical power of 80%, the minimum difference between the means was 3.2 ± 0.53 with an effect size *f* corresponding to 1.48 for QMT, and a sample size of 12 patients was required in each group. In addition, 3 patients (≈ 20%) were included due to potential sample loss, resulting in 45 patients divided into three groups.

### 2.7 Statistical Analysis

Data were analyzed using the GraphPad Prism 5 statistical software (GraphPad Software Inc., San Diego, CA, USA). The normality of the variables was assessed using the Shapiro‐Wilk test. Continuous variables were reported as means ± standard deviation (SD) and 95% confidence intervals (95% CI), or median and interquartile range (IQR 25–75th percentile) values, and the categorical variables were reported as absolute frequencies and percentages. The independent Student's *t*‐test was used to compare the baseline data for the outcome variables (QMT and MRC) between the groups. The Student's *t*‐test for paired samples was used to compare the data before and after the intragroup intervention. Between‐group comparisons were performed using two‐way ANOVA followed by Bonferroni post hoc. The variation between the first 24 h of MV (pre‐intervention) and ICU discharge (post‐intervention) was considered for analysis. The effect size was calculated using Cohen's d. The significance level was set at 5% for all analyses (*p* < 0.05).

## 3 Results

**FIGURE 1:**
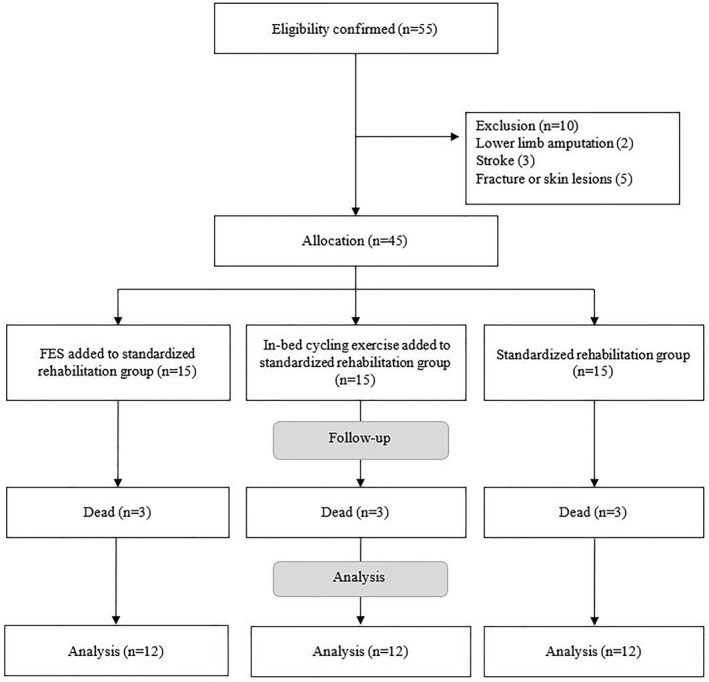
Study flow chart.

Of the 55 eligible patients, 10 were excluded due to not meeting the inclusion criteria, then 45 were divided into three groups: NMES added to standardized rehabilitation group (*n* = 15), in‐bed cycling exercise added to standardized rehabilitation group (*n* = 15) or to standardized rehabilitation group (*n* = 15). Subsequently, three patients in each group died before the study was completed. Thus, the final sample consisted of NMES added to standardized rehabilitation group (*n* = 12), in‐bed cycling exercise added to standardized rehabilitation group (*n* = 12) or to standardized rehabilitation group (*n* = 12) (Figure 1). At baseline, the characteristics of the participants and the outcome variables (QMT and MRC) were similar in all three groups (Table 1). During in‐bed cycling exercise added to standardized rehabilitation group, two adverse events were observed in three individual patients: increased respiratory rate and decreased SpO_2_. None of the patients in the NMES added to standardized rehabilitation group or the standardized early rehabilitation group experienced adverse events.

**TABLE 1:** Demographic and clinical characteristics of patients participating in the study.

Characteristics	Standardized rehabilitation group (*n* = 12)	In‐bed cycling exercise added to standardized rehabilitation group (*n* = 12)	NMES added to standardized rehabilitation group (*n* = 12)
Age, mean (SD), years	48.6 ± 20.3	46.7 ± 19.9	46.6 ± 14.7
Male patients, *n* (%)	11 (91.6)	10 (83.3)	6 (54.5)
BMI kg/m^2^, mean (SD)	25.7 ± 6.2	25.6 ± 5.9	25.7 ± 4.9
APACHE II score, mean (SD)	18.1 ± 6.2	16 ± 5.8	15.2 ± 5.9
SOFA score, mean (SD)	7 (3)	8 (3)	7 (3)
Primary diagnosis at ICU admission, *n* (%)			
Cardiovascular	0 (0.0)	1 (8.3)	1 (9.0)
Gastrointestinal (non‐surgical)	1 (8.3)	2 (16.6)	2 (18.1)
Neurological	2 (16.6)	5 (41.6)	6 (54.5)
Respiratory	6 (50)	2 (16.6)	2 (18.1)
Metabolic	3 (25)	2 (16.6)	0 (0.0)
ICU length of stay, days, mean (SD)	10 (5)	11 (5)	10 (5)
Duration of MV, days, mean (SD)	8 (4)	8 (3)	8 (3)
Time to first assessment by the MRC, days, median (IQR)	2.5 (2.5–3)	2 (2–3)	2 (2–3)
Time to first assessment by the ultrasound, days, median (IQR)	1 (1–2)	1 (1–2)	1 (1–2)
Time to initiate exercise protocol, days, median (IQR)	1 (1–2)	1 (1–2.5)	1 (1–2)
Number sessions, days, median (IQR)	17 (16–20)	10 (10–12.5)	10 (10–12.5)
Medications, *n* (%)			
Neuromuscular blocking agents	10 (83.3)	10 (83.3)	10 (83.3)
Glucocorticoids	8 (66.6)	8 (66.6)	8 (66.6)
Vasoactive agents	10 (83.3)	10 (83.3)	10 (83.3)
Outcome variables			
QMT (cm)	2.5 ± 0.8	2.9 ± 0.5	3.1 ± 0.7
MRC (score)	46.2 ± 9.7	40.6 ± 2.6	40.3 ± 1.3

*Note:* Data are expressed as mean (SD), *n* (%) and as median and interquartile range (IQR).

Abbreviations: APACHE II = Acute Physiology and Chronic Health Evaluation II, APACHE II = Acute Physiology and Chronic Health Evaluation II; BMI = Body mass index, ICU = Intensive care unit, MRC = Medical Research Council, MV = Mechanical ventilation, NMES = functional electrical stimulation; QMT = Quadriceps Muscle Thickness, SOFA = Sequential Organ Failure Assessment.

Throughout the intervention, all participants in the in‐bed cycling exercise group completed the sessions exclusively in passive mode. At patient awake (RASS 0), the MOTOmed letto2 device occasionally detected brief active movements; however, these were not sustained. Consequently, no participant achieved active cycling, and all sessions were performed in passive mode only.

There was a significant reduction in QMT from pre‐to post‐intervention in the standardized rehabilitation group (2.5 ± 0.8 cm vs. 1.9 ± 0.8 cm; 95% CI: −0.9 to −0.3; *p* = 0.001). However, there was no significant change in the QMT in the in‐bed cycling exercise added to standardized rehabilitation group (2.9 ± 0.5 cm vs. 3.0 ± 0.5 cm; 95% CI: −0.1 to 0.4; *p* = 0.315) and in the NMES added to standardized rehabilitation group (3.1 ± 0.7 cm vs. 2.8 ± 0.8 cm; 95% CI: −0.7 to 0.1; *p* = 0.158) (Table 2). Compared with the standardized rehabilitation group, we observed that the in‐bed cycling exercise group (1.1 cm; 95% CI: 0.3 to 1.8; *p* < 0.001; *d* = 0.989) and NMES group (0.9 cm; 95% CI: 0.1 to 1.6; *p* < 0.01; *d* = 0.735) preserved QMT (Table 3). There was no difference in the QMT between the in‐bed cycling exercise added to the standardized rehabilitation group and NMES added to standardized rehabilitation group after the intervention (−0.2 cm; 95% CI: −1.0 to 0.6; *p* > 0.05; *d* = 0.627) (Table 3).

**TABLE 2:** Intra‐group comparison for the outcomes quadriceps muscle thickness and overall muscle strength.

	Standardized rehabilitation group (*n* = 12)				In‐bed cycling exercise added to SR group (*n* = 12)				NMES added to SR group (*n* = 12)			
Variable	Pre mean ± SD	Post mean ± SD	Difference in mean (CI 95%)	*p‐*value^a^	Pre mean ± SD	Post mean ± SD	Difference in mean (CI 95%)	*p‐*value^a^	Pre mean ± SD	Post mean ± SD	Difference in mean (CI 95%)	*p‐*value^a^
QMT (cm)	2.5 ± 0.8	1.9 ± 0.8	−0.6 (−0.9 to −0.3)	0.001^b^	2.9 ± 0.5	3.0 ± 0.5	0.1 (−0.1–0.4)	0.315	3.1 ± 0.7	2.8 ± 0.8	−0.3 (−0.7 to 0.1)	0.158
MRC (score)	46.2 ± 9.7	46.3 ± 15.7	0.1 (−9.8 to 9.5)	0.970	40.6 ± 2.6	47.7 ± 0.9	7.1 (5.4–8.9)	< 0.001^b^	40.3 ± 1.3	45.5 ± 2.3	5.2 (−3.6–6.7)	< 0.001^b^

*Note:* Data are expressed as mean (SD).

Abbreviations: MRC: Medical Research Council; NMES: neuromuscular electrical stimulation; QMT: quadriceps muscle thickness; SR: Standardized Rehabilitation.

^a^ Intragroup comparison: Student *T* test.

^b^ Significant difference (*p* < 0.05).

**TABLE 3:** Inter‐group comparison for the outcomes quadriceps muscle thickness and overall muscle strength.

Variable	Groups	Interaction effect (group vs time) difference between groups (CI 95%)	*p‐*value^a^	Effect size
QMT (cm)	In‐bed cycling exercise added to SR versus SR	1.1 (0.3–1.8)	< 0.001^b^	0.989
	NMES added to SR versus SR	0.9 (0.1–1.6)	< 0.01^b^	0.735
	NMES added to SR versus In‐bed cycling exercise added to SR	−0.2 (−1.0 to 0.6)	> 0.05	0.627
MRC (score)	In‐bed cycling exercise added to SR versus SR	1.4 (−7.2–10.1)	> 0.05	0.638
	NMES added to SR versus SR	−0.8 (−9.6 to 8.1)	> 0.05	0.471
	NMES added to SR versus In‐bed cycling exercise added to SR	−2.2 (−11.1 to 6.6)	> 0.05	0.654

Abbreviations: MRC: Medical Research Council, NMES: neuromuscular electrical stimulation, QMT: quadriceps muscle thickness, SR: Standardized Rehabilitation.

^a^ Comparison between groups: two‐way ANOVA followed by Bonferroni post hoc.

^b^ Significant difference (*p* < 0.05).

Regarding the MRC score, a significant increase was observed in the in‐bed cycling exercise added to standardized rehabilitation group (40.6 ± 2.6 vs. 47.7 ± 0.9; 95% CI: 5.4 to 8.9; *p* < 0.001) and in the NMES added to standardized rehabilitation group (40.3 ± 1.3 vs. 45.5 ± 2.3; 95% CI: −3.6 to 6.7; *p* < 0.001) after the intervention. However, the MRC score in the standardized rehabilitation group remained unchanged (46.2 ± 9.7 vs. 46.3 ± 15.7; 95% CI: −9.8 to 9.5; *p* = 0.970) (Table 2). No differences were observed among the groups after the intervention (Table 3).

## 4 Discussion

Our study conducted with critically ill patients recruited in the first 24 h of MV suggested that NMES and in‐bed cycling exercise added to standardized rehabilitation provided additional benefit to preserve QMT. In addition: (1) the standardized rehabilitation group showed a decline in QMT; (2) the global muscle strength, assessed by the MRC score, showed an increase only in NMES and in‐bed cycling exercise added to standardized rehabilitation groups at ICU discharge.

Previous studies have reported that early mobilization strategies in the ICU have generated a lot of interest as a potential intervention to prevent muscle wasting and enhance functional recovery in critically ill patients (Engel and Brummel 2024; Anekwe et al. 2020). In this sense, especially considering that muscle wasting starts early and every 1% loss of QMT over the first week of critical illness was independently associated with a higher 1‐year mortality (Lee et al. 2021), our results suggest that early implementation of in‐bed cycling exercise and NMES as a promising modalities of rehabilitation to attenuate loss of muscle mass, which may be beneficial for patient recovery (Chapple et al. 2022).

Among the effective, safe and low‐cost early interventions, NMES has been considered a promising treatment for critically ill patients (García‐Pérez‐de‐Sevilla and Sánchez‐Pinto 2023; Silva et al. 2019). Our results are consistent with a recent randomized clinical trial that demonstrated that an early NMES protocol can preserve muscle thickness in patients with traumatic brain injury (Vieira et al. 2023). As demonstrated in our study, the authors also observed a decrease in muscle thickness in the control group (usual care). The attenuation of muscle atrophy may be explained, at least speculatively, by the ability of NMES to enhance peripheral blood flow, promote muscle protein synthesis and facilitate a favorable balance between pro‐ and anti‐inflammatory cytokines, possibly through the direct stimulation of muscle fibers (Winkelman 2004; Friedrich et al. 2015). Nevertheless, pro‐ and anti‐inflammatory cytokines were not assessed in the present study.

In the present study, we suggested that in‐bed cycling exercise added to standardized rehabilitation also effectively preserved muscle thickness. This finding is consistent with a previous randomized clinical trial conducted with adult patients within the first 72 h of ICU admission, which demonstrated that an early mobilization program, based on twice daily sessions of both manual mobilization and 30‐min passive/active cycling therapy, preserved the cross‐sectional area of the quadriceps femoris (Hickmann et al. 2018). However, the study conducted by Hickmann et al. (2018) differs from our study for the following reasons: (1) authors recruited only septic shock patients; (2) muscle fiber was measured by muscle biopsy; and (3) patients were recruited within the first 72 h of ICU admission. In this sense, our findings are clinically relevant because critically ill patients experience early severe inflammation‐induced increased muscle protein breakdown, metabolic alterations, and loss of muscle mass (E. E. França et al. 2017; E. E. T. França et al. 2020) and we hypothesized that the timing of early in‐bed cycling exercise ‐ initiated in the first 24 h of MV ‐ may counteract acute critical illness‐induced catabolism and could attenuate muscle atrophy. In a previous study, our research group showed that a single 20‐min session of passive in‐bed cycling exercise seems to have a positive effect on the inflammatory response in critically ill patients (Carvalho et al. 2020).

Our study found an increase in global muscle strength in patients who underwent NMES and in‐bed cycling exercise. These results are consistent with a recent meta‐analysis that showed that both NMES and in‐bed cycling increased muscle strength (García‐Pérez‐de‐Sevilla and Sánchez‐Pinto 2023). Another meta‐analysis conducted by M. Liu et al. (2020) showed that the early implementation of NMES can effectively improve the muscle strength and prevent the occurrence of ICU‐AW to a certain extent. It is noteworthy that according to the commonly used threshold to diagnose ICU‐AW, our patients showed MRC score ≤ 48 points at ICU‐discharge. The clinical interpretation of these findings warrants consideration. Hough et al. (2011) demonstrated that the MRC score is insufficient for early detection of ICU‐AW and may be unreliable during critical illness. Although we included only patients with sufficient cognitive ability to cooperate with strength testing, it is important to consider the hypothesis of persistent delirium in our sample. B. A. Connolly et al. (2013) suggested that alternative non‐volitional strategies are required to evaluate and monitor muscle function in the early stages of critical illness.

Despite the relevance of our results, some limitations should be considered. First, our study has a quasi‐experimental design. Second, because our study aimed to investigate the effects of early NMES and in‐bed leg cycling, the duration of both interventions was relatively short. Thus, our findings should be confirmed in large randomized clinical trials with longer follow‐up. Third, some patients received corticosteroids and neuromuscular blocking agents; notably, the use of neuromuscular blocking agents during ICU stay is a known risk factor for developing ICU‐AW (Yamada et al. 2023). Future studies should be directed toward addressing these limitations.

In conclusion, this study suggests that early NMES and in‐bed cycling exercise added to standardized rehabilitation preserve quadriceps muscle thickness in critically ill patients. Larger trials are needed to evaluate the effectiveness of NMES and in‐bed cycling exercise on QMT and global muscle strength in ICU patients.

## 5 Implications of Physiotherapy Practice

Considering that muscle wasting often begins rapidly after ICU admission and progressively worsens over time (Nakanishi et al. 2019), the potential clinical practice implications of our study suggested that early interventions ‐ NMES and in‐bed cycling exercise ‐ combined with standardized rehabilitation provided a protective effect on muscle thickness. In our study, intervention protocols were implemented at the latest on the second day of ICU admission, which may have attenuated muscle atrophy and may be beneficial for patient recovery. In a recent state‐of‐the‐art review, Hiser et al. (2025) highlighted that the timing of physical therapy initiation is another important factor in the ICU setting. According to Hodgson et al. (2016), when interventions are truly started early (e.g., within 48 h of ICU admission), the focus of mobilization is preserved muscle mass. Our findings also support the use of early NMES, in‐bed cycling exercise, and bedside ultrasound of quadriceps muscle layer thickness as safe and feasible strategies in the ICU.

## Author Contributions

Bruna Scherer Lorenzoni, Marina Torres Machado and Michele Forgiarini Saccol and contributed to the study concept, design, data acquisition and administrative, technical, and material support, drafting the manuscript and critical revision of the manuscript. Maurício Tatsch Ximenes Carvalho, Tamires Daros dos Santos and Dannuey Machado Cardoso contributed to the data analysis and interpretation, drafting the manuscript, critical revision of the manuscript and statistical analysis. João Pedro Martins de Albuquerque, Helena Biermann Pereira, and Rafaela Bassan Bortoluzi contributed to the drafting of the manuscript, critical revision of the manuscript and statistical analysis. Isabella Martins de Albuquerque contributed to the study concept, design, data acquisition and administrative, technical and material support, drafting the manuscript, critical revision of the manuscript, statistical analysis and study supervision.

## Ethics Statement

This study was approved by the local Institutional Review Board (project number: 1.067.797).

## Consent

Written informed consent was obtained from all included patients in accordance with the Helsinki Declaration.

## Conflicts of Interest

The authors declare no conflicts of interest.

## Permission to Reproduce Material From Other Sources

The authors have nothing to report.

## Data Availability

Anonymized data are available on request.
